# Social Media – Chancen und Risiken für die Rheumatologie

**DOI:** 10.1007/s00393-022-01201-9

**Published:** 2022-04-08

**Authors:** I. Haase, J. Mucke, D. Vossen, J. Knitza, N. Ruffer, M. Zeeck, M. Krusche

**Affiliations:** 1 Arbeitsgemeinschaft Junge Rheumatologie, Deutsche Gesellschaft für Rheumatologie e. V., Berlin, Deutschland; 2 Poliklinik, Funktionsbereich und Hiller-Forschungszentrum für Rheumatologie, Universitätsklinik Düsseldorf, Heinrich-Heine-Universität, Düsseldorf, Deutschland; 3 Rheinisches Rheumazentrum Meerbusch, St. Elisabeth Hospital, Meerbusch, Deutschland; 4 Medizinische Klinik 3 – Rheumatologie und Immunologie, Universitätsklinikum Erlangen, Friedrich-Alexander-Universität Erlangen-Nürnberg (FAU), Erlangen, Deutschland; 5 Klinik für Rheumatologie und Immunologie, Klinikum Bad Bramstedt, Bad Bramstedt, Deutschland; 6 Sektion für Rheumatologie und Entzündliche Systemerkrankungen, Universitätsklinikum Eppendorf (UKE), Martinistr. 52, 20246 Hamburg, Deutschland

**Keywords:** Soziale Netzwerke, Plattform, Verlinkung, Influencer, Crowdsourcing, Education, Awareness, Datenschutz, Persönlichkeitsrechte, Social networks, Platform, Link, Influencer, Crowd sourcing, Education, Awareness, Data protection, Personal rights

## Abstract

Die Nutzung von sozialen Medien (Social Media) und sozialen Netzwerken (Social Networks) hat in den letzten Jahren stark zugenommen. Sie gewinnen als Informationskanäle sowohl im privaten als auch beruflichen Kontext immer mehr an Bedeutung. Auch in der Medizin werden Social Media bereits vielfältig eingesetzt. So sind Fachgesellschaften und Interessenverbände immer stärker in den sozialen Netzwerken vertreten. Durch die breite Nutzung und große Reichweite der Netzwerke ergeben sich neue Möglichkeiten auch für das Fach der Rheumatologie. Dieser Übersichtsartikel gibt einen Überblick über die Charakteristika einiger großer Social-Media-Plattformen und untersucht bisherige Publikationen aus diesem Themengebiet im Rahmen einer systematischen Analyse. Weiterhin werden Vorteile, aber auch potenzielle Risiken, die bei der Nutzung entstehen können, beschrieben.

**Zusatzmaterial online:**

Zusätzliche Informationen sind in der Online-Version dieses Artikels (10.1007/s00393-022-01201-9) enthalten.

## Abstract (en)

The use of social media and social networks has increased significantly in recent years. They are becoming progressively more important as information channels in private and professional contexts. Also, in medicine, social media are already being used in a variety of ways. For example, professional societies and patient interest groups are being increasingly represented in social networks. The broad use and wide audience of these networks offer new opportunities for the field of rheumatology. This review article provides an overview of the characteristics of some major social media platforms and systematically analyses the existing publications in the context of rheumatology. Furthermore, advantages, but also potential risks that may arise due to social media use are being addressed.

Die Nutzung und die Relevanz von sozialen Netzwerken (Social Networks) und sozialen Medien (Social Media) haben in den letzten Jahren stark zugenommen. Netzwerkanbieter wie Facebook oder Google gehören aktuell zu den wertvollsten und einflussreichsten Unternehmen der Welt. Im Jahr 2021 nutzen weltweit schätzungsweise 4,4 Mrd. Menschen soziale Medien [1]. In Deutschland sind es im selben Jahr ca. 66 Mio. Nutzer*innen [2].

Soziale Medien gewinnen dabei immer mehr als Informationskanäle sowohl im privaten als auch beruflichen Kontext an Bedeutung. Viele Menschen verbringen inzwischen mehr Zeit mit sozialen Medien als mit analogen Nachrichtenkanälen wie Printmedien oder Fernsehen. Auch in der Medizin werden Social Media bereits vielfältig eingesetzt. Akteure wie Kliniken, Fachgesellschaften und Patientenverbände implementieren sie in ihre öffentliche Kommunikation, aber gerade auch Einzelpersonen generieren Inhalte und erreichen hier ein großes Publikum. Eine EMEUNET-Analyse konnte 2017 zeigen, dass unter den jungen Rheumatolog*innen (Alter bis 39 Jahre) 71 % der Befragten aktiv mindestens eine Social-Media-Plattform im beruflichen Kontext nutzten [3].

Möchte man Social Media effektiv für die Verbreitung eigener Inhalte verwenden, gilt jedoch zu beachten, dass es zwischen den Plattformen teilweise deutliche Unterschiede in den Eigenschaften und dem jeweiligen Nutzungsverhalten gibt.

Dieser Artikel gibt einen Überblick über die Charakteristika einiger großer Social-Media-Plattformen. Weiterhin sollen Vorteile, aber auch potenzielle Risiken, die durch deren Nutzung entstehen können, aufgezeigt werden.

## Begrifflichkeiten und Social-Media-Plattformen

Die Begriffe soziale Medien und soziale Netzwerke werden im Alltag oft synonym verwendet. In Fachkreisen sind aktuell folgende Definitionen verbreitet: Soziale Medien sind webbasierte Anwendungen und Plattformen, die es ermöglichen, Nutzer*innen-generierte Inhalte zu verbreiten und sich über diese mit anderen Nutzer*innen auszutauschen. Welche Inhalte den individuellen Anwender*innen angezeigt werden, bestimmt in den meisten Fällen ein Algorithmus, der, basierend auf den bisher besuchten Inhalten, eine Auswahl ähnlicher Beiträge trifft. Als Subtypen zählt man zu den sozialen Medien neben den sozialen Netzwerken wie Facebook beispielsweise auch Blogs, Plattformen zum Teilen von Videos oder Webseiten zur Bewertung von Produkten und Dienstleistungen. Eine klare Einordnung wird zum Teil durch eine Multifunktionalität der Plattformen erschwert. Der Fokus sozialer Netzwerke liegt darauf, Verbindungen und Dialog zwischen Nutzer*innen zu ermöglichen, die sich kennen oder gemeinsame Interessen haben.

Im Bereich der sozialen Netzwerke haben sich in den letzten Jahren einige große Anbieter etabliert. Die Plattformen unterscheiden sich teilweise sehr in ihren technischen Interaktionsmöglichkeiten, ihren Inhalten und Zielgruppen. Erfolgreiche Funktionen, die zunächst ein Alleinstellungsmerkmal einer Plattform bilden, werden in der Folge aber auch immer wieder von anderen Diensten übernommen (z. B. Like-Button).

**Figure 1:**
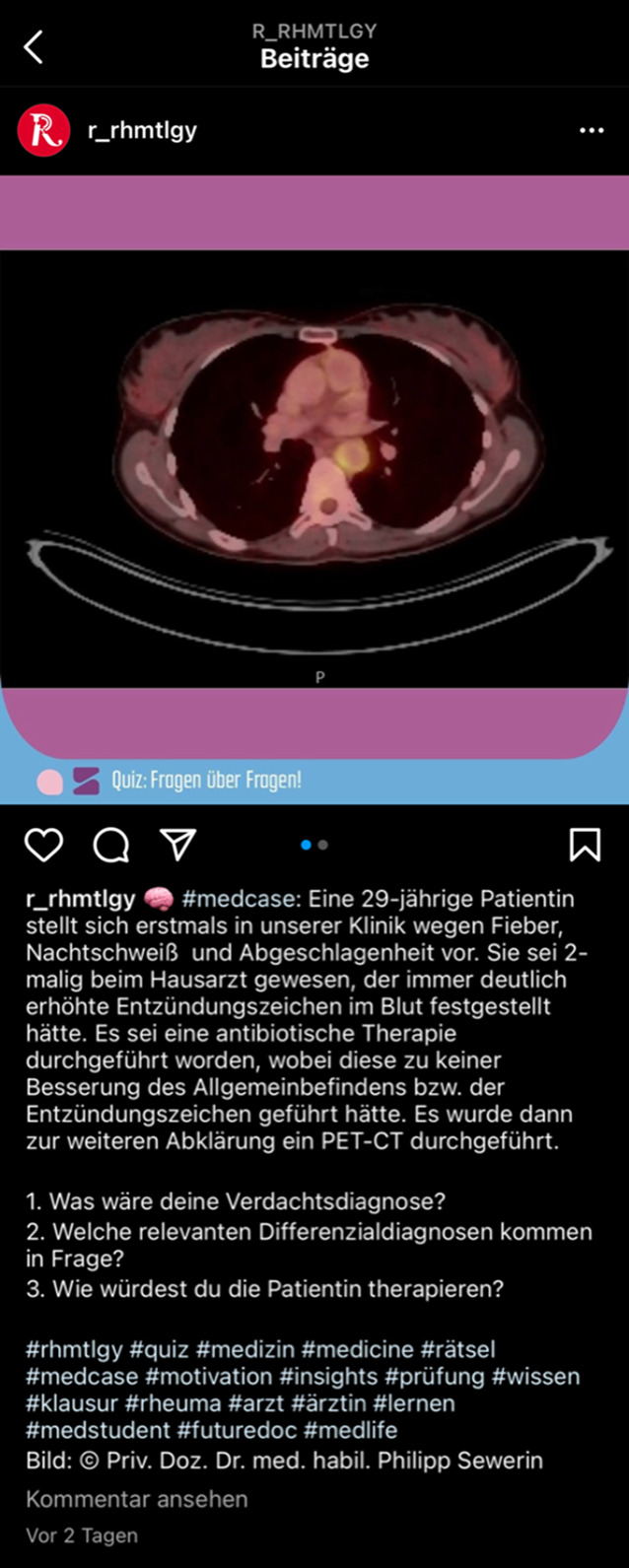

**Figure 2:**
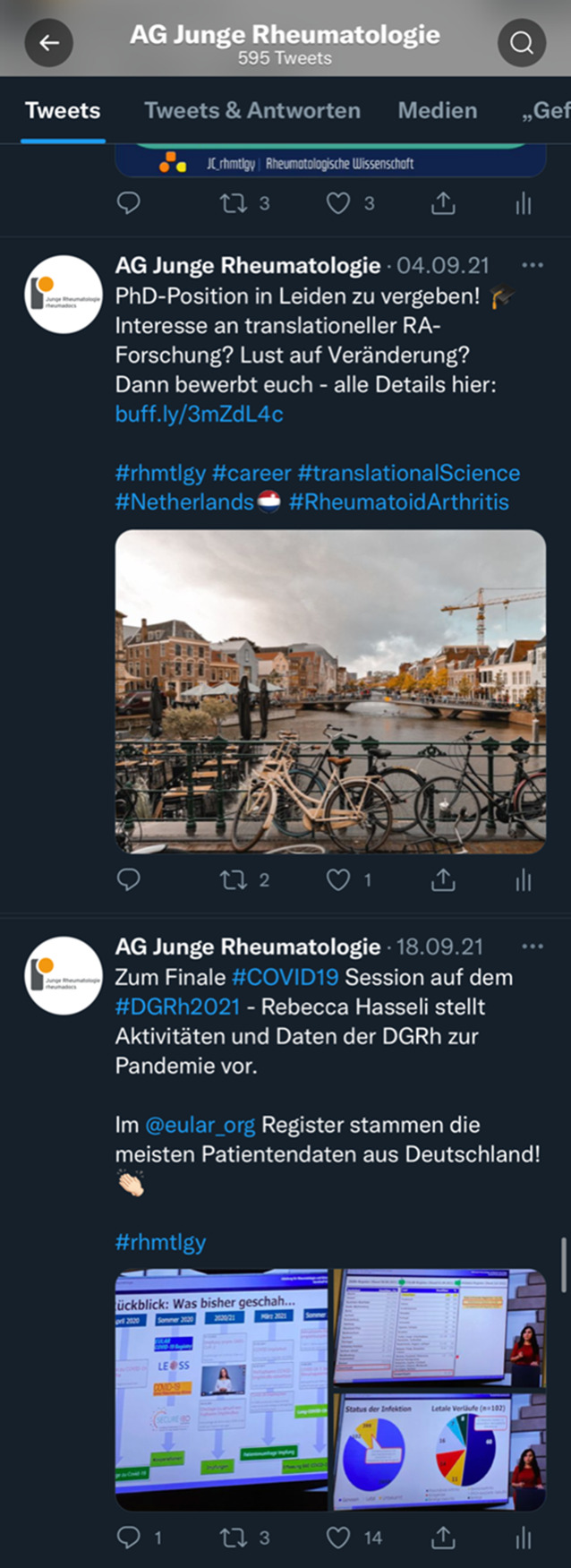

Facebook stellt eines der am längsten etablierten Netzwerke dar und ist nahezu bei allen Altersgruppen verbreitet [4]. Instagram ist als Plattform sehr visuell orientiert und wird verstärkt von einer jüngeren Zielgruppe genutzt (Abb. 1). Demgegenüber fokussiert sich die Plattform LinkedIn auf eine Vernetzung von Personen mit überlappenden beruflichen Interessen, sodass sich auch die Profile und Inhalte thematisch hieran orientieren. Twitter als prominenter Kurznachrichtendienst wird als Austauschplattform sowohl für private als auch berufliche Inhalte verwendet [5]. Auf internationaler Ebene wird Twitter breitflächig von Wissenschaftler*innen für den Austausch genutzt (Abb. 2). Einen Überblick über einige wichtige soziale Medien gibt Tab. 1 ohne Anspruch auf Vollständigkeit. In Tab. 2 findet sich eine Hyperlink-Sammlung der im Beitrag thematisierten Angebote.

**Table 1:** 

Plattform	Anbieter	Nutzer*innen-Zahl (in Mio.)	Aktive Nutzer*innen/Monat (in Mio.)	Kurzbeschreibung
Facebook	Meta Platforms, Inc.	–	2895	Netzwerk zur Erstellung von (ausführlichen) privaten Profilen durch Teilen von Statusmeldungen, Text, Fotos, Videos. Newsfeed („Timeline“) dominiert die Ansicht – mit geteilten Inhalten vernetzter Nutzer*innen und abonnierter Gruppen, Veranstaltungen u. Ä.
				Vernetzung über das Knüpfen von „Freundschaften“; Abonnieren von Seiten; Kommentieren; Markierung mit „Gefällt mir“; Facebook-Messenger ermöglicht Live Chats
Instagram	Meta Platforms, Inc.	–	1435	Netzwerk zum Teilen von Foto- und Videoinhalten mit einer Follower-Gemeinschaft (Abonnent*innen). Sowohl das eigene Profil als auch der Feed sind stark bildgeprägt. Ermöglicht Nutzer*innen den Austausch via Kurzkommentar, Likes („Gefällt mir“) und Privatnachrichten. Primär für mobile Endgeräte entwickelt
Twitter	Twitter Inc.	Ca. 330 (2019)	206*	Microbloggingdienst zur Verbreitung telegrammartiger Kurznachrichten (max. 280 Zeichen) mit der Möglichkeit, anderen Nutzer*innen zu folgen und deren Beiträge zu favorisieren. Textlastig, Teilen von Bildern und Videos aber möglich. Dank hoher Aktualität und Schnelligkeit als Kurznachrichtendienst- und Mitteilungsplattform etabliert. Nur wenige Informationen auf dem eigenen Profil, Privatnachrichten sind möglich
LinkedIn	Microsoft Corporation	756	310 (2019)	Netzwerk mit Fokus auf Karrierenetzwerken; zur Pflege von Geschäftskontakten und fachlichem Austausch. Profile bestehen aus aktuellem Lebenslauf und beruflichem Werdegang. Ermöglicht Interaktion über Kommentare, Likes und von Nutzer*innen erstellte Blogartikel („long form posts“)
Youtube	Google LLC	–	2000	Videoplattform mit der Möglichkeit zur Erstellung eines eigenen Kanals, den Nutzer*innen abonnieren können. Nutzer*inneninteraktion erfolgt über eine Kommentarfunktion und Likes. Vernetzung steht eher im Hintergrund. Inhalte breit gefächert, z. B. auch aufgezeichnete Vorlesungen, Vorträge, Wissensvermittlung

Definitionen für monatlich aktive Nutzer*innen (MAN):

Twitter *monetarisierbare *MAN: Personen, Organisationen, andere Konten, die an einem bestimmten Tag bei Twitter eingeloggt sind und für die Anzeigen geschaltet werden können

Instagram MAN: registrierte und eingeloggte Nutzer*innen, die innerhalb von 30 Tagen ab dem Messdatum die App oder Webanwendung von Instagram besuchen

Facebook: definiert als registrierter und eingeloggter Nutzer*in von Facebook, der die Website in den letzten 30 Tagen zum Zeitpunkt der Messung über eine Mobilgeräteanwendung oder einen Webbrowser besucht hat

**Table 2:** 

Angebot	Hyperlink
COVID-19 Global Rheumatology Alliance	www.rheum-covid.org
DGRh COVID19 Register	www.covid19-rheuma.de
Doc2Doc	www.doc2doc.ch
Doctors Hangout	www.doctorshangout.com
Doximity	www.doximity.com
Facebook	www.facebook.com
Instagram	www.instagram.com
LinkedIn	www.linkedin.com
maiLab (YouTube Kanal)	www.youtube.com/mailab
Medical Directors Forum	www.medicaldirectorsforum.com
Sermo	www.sermo.com
Snapchat	www.snapchat.com
Streamedup!	www.streamed-up.com
Student Doctor Network	www.studentdoctor.net
TikTok	www.tiktok.com
Twitter	www.twitter.com
YouTube	www.youtube.com

Durch die steigende Popularität und Nutzung von Social Media sind auch verschiedene Fachgesellschaften, Patientenverbände, aber auch wissenschaftliche Journale zusehends auf den Kanälen aktiv (Tab. 3 und 4).

**Table 3:** 

Journal	Kennzahl Reichweite				
	Facebook (Likes)	Instagram (gefolgt von)	LinkedIn (gefolgt von)	Twitter (gefolgt von)	Youtube (Abonnenten)
*Nature Reviews of Rheumatology*^a^	449.189	164.000	137.479	12.600	8840
*Annals of the Rheumatic Diseases*^b^	6472	–	–	7692	437
*Arthritis and Rheumatology*^c^	–	–	–	5861	–
*Rheumatology*^d^	3558	–	–	7621	–
*Therapeutic Advances in Musculoskeletal Disease*	*SAGE gemeinsame Profile*^*g*^				
*Osteoarthritis and Cartilage*^e^	–	–	–	2189	–
*Seminars in Arthritis and Rheumatism*	–	–	–	1198	–
*Arthritis Research and Therapy*	–	–	–	3504	–
*Arthritis Care and Research*^c^	–	–	–	25.000	–
*Current Opinion in Rheumatology*	–	–	–	882	–
*Current Rheumatology Reports*^e^	–	–	–	1382	–
*Joint Bone Spine*^f^	*Elsevier gemeinsame Profile*^h^				
*Rheumatology and Therapy*	–	–	–	979	–
*The Journal of Rheumatology*	4730	–	761	914	377
*Clinical and Experimental Rheumatology*	–	–	–	925	–
*Rheumatic Disease Clinics of North America*^f^	*Elsevier gemeinsame Profile*^h^				
*Scandinavian Journal of Rheumatology*	–	–	–	–	–
*Best Practice & Research in Clinical Rheumatology*^f^	*Elsevier gemeinsame Profile*^h^				
*Pediatric Rheumatology*	–	–	–	–	–
*Clinical Rheumatology*	1210	–	–	–	–
^g^*SAGE gemeinsame Profile*	14.048	3496	46.286	7195	10.700
^h^*Elsevier gemeinsame Profile*	304.708	13.900	402.999	97.400	9200

^a^Facebook Seite für Nature insg.; LinkedIn und Instagram Profile von Nature Research verfügbar; Profil spezifisch von *Nature Reviews of Rheumatology* nur auf Twitter; Youtube gemeinsames Profil Nature Portfolio

^b^Beide Profile (auf Facebook und Twitter) sind gemeinsame Profile für die Zeitschriften *Annals of the Rheumatic Diseases* und *Rheumatic and Muskuloskeletal Diseases Open*

^c^Nachrichten und Updates über die Zeitschriften *Arthritis Care and Research* und *Arthritis and Rheumatology* sind nur auf dem Twitter-Profil der Zeitschriften des American College of Rheumatology verfügbar

^d^Gemeinsames Twitter-Konto für *Rheumatology* und *Rheumatology Advances in Practice*; Nachrichten und Updates *Rheumatology* sind nur auf den Seiten der Britischen Gesellschaft für Rheumatologie verfügbar

^e^Nachrichten und Updates über alle Springer Current Reports Journale sind nur auf einem gemeinsamen Twitter-Profil mit der genannten Folgerzahl verfügbar

^f^Nachrichten und Updates über die Journale (*Joint Bone Spine, Rheumatic Disease Clinics of North America, Best Practice & Research in Clinical Rheumatology*) sind nur auf den gemeinsamen Profilen von Elsevier verfügbar

**Table 4:** 

Fachgesellschaft/Patient*innenenorganisation	Kennzahl Reichweite				
	Facebook (Likes)	Instagram (gefolgt von)	LinkedIn (gefolgt von)	Twitter (gefolgt von)	Youtube (Abonnenten)
Deutsche Gesellschaft für Rheumatologie (DGRh)	538	–	–	–	–
European League against Rheumatism (EULAR)	12.612	2734	4381	15.700	3140
British Society for Rheumatology	3671	–	2004	13.800	314
American College of Rheumatology	23.794	1228	8039	27.400	2480
AGJR – rheumadocs	658	407	–	341	–
EMEUNET	2276	–	–	5375	144
Deutsche Rheumaliga	24.650	5347	–	1402	6530
EULAR Young PARE	1032	786	–	2085	–
EULAR PARE	–	–	–	–	3140
The Lupus Initiative (ACR)	2842	1138	243	10.100	22

## (Virale) Verbreitung von Inhalten und Medical Influencer

Beiträge (*Tweets, Posts*) werden in den sozialen Medien häufig mit Schlagworten (*Hashtags, #*) versehen, die Inhalte thematisch verknüpfen und per Suchfunktion auffindbar machen. Gleichzeitig können in einem Beitrag (meist mittels *@*) andere Nutzer*innen verlinkt werden, wodurch bei diesen Aufmerksamkeit generiert wird. All dies führt – auch aufgrund von oben bereits angesprochenen Algorithmen – zu einer verstärkten Verbreitung derart gekennzeichneter Inhalte. Von einer viralen Verbreitung spricht man, wenn Inhalte innerhalb kürzester Zeit eine große Anzahl an Nutzer*innen auf einer Plattform erreichen.

Als *Influencer* werden hoch aktive Nutzer*innen mit großer Reichweite innerhalb eines sozialen Netzwerkes bezeichnet. Sie werden durch ihre Abonnent*innen (*Follower*) häufig als vertrauenswürdige Expert*innen angesehen. Durch diese Eigenschaften können sie mit ihren Botschaften eine große Anzahl an Abonnent*innen erreichen und beeinflussen. Als *Medical Influencer* treten Ärzt*innen, Forschende oder Patient*innen auf und erreichen durch eine solche Funktion als Multiplikator*in teilweise Millionen von Nutzer*innen [5].

Insbesondere auf Twitter bewerben akademisch tätige Rheumatolog*innen beispielsweise eigene oder für sie bedeutsame Publikationen, informieren über digitale Lernangebote und Ressourcen („from twitter to bedside“), initiieren kollektive Projekte und teilen klinische Erfahrungen. Von großer Bedeutung ist die Tatsache, dass Inhalte dabei nicht zwangsläufig wissenschaftlich geprüft sind und somit Raum für Falschinformationen und unethisches Verhalten besteht.

## Systematische Analyse Social-Media-assoziierter Publikationen (Stand 01.02.2022)

Neben ihrer Kommunikationsfunktion sind Social Media inzwischen sowohl Gegenstand als auch Werkzeug der rheumatologischen Forschung.

Zur Analyse rheumatologischer Publikationen mit Bezug zu Social Media wurde eine systematische Literaturrecherche auf MEDLINE (via PubMed) von 2 unabhängigen Untersucher*innen durchgeführt. Eine detaillierte Darstellung der Suchstrategie findet sich im Online-Zusatzmaterial.

Insgesamt konnten 217 Publikationen auf MEDLINE (via PubMed) identifiziert werden, von denen 106 in die Analyse eingeschlossen werden konnten. In 40,5 % (43/106) der Publikationen wurden Social Media zur Verteilung bzw. Bewerbung von Studien oder Umfragen verwendet; 39,6 % (42/107) der Arbeiten waren Inhaltsanalysen („content analysis“) von Social-Media-Daten zu rheumatologischen Themen; 12,2 % (13/106) der Publikationen waren Übersichtsarbeiten (Reviews), die sich mit Social Media in der Rheumatologie beschäftigten. In 8,4 % (9/106) wurde in übergeordneten Analysen auch über den Einfluss oder die Nutzung von Social Media im Kontext von rheumatologischen Themengebieten berichtet.

Die publizierten Inhaltsanalysen untersuchten am häufigsten Inhalte der Videoplattform YouTube. Inhalte von oder für Patient*innen wurden in den Publikationen häufiger analysiert als solche für medizinisches Personal. Wenige Publikationen beschränkten sich auf einzelne rheumatologische Erkrankungen. Am häufigsten waren jedoch die Themen „COVID-19“ und „rheumatoide Arthritis“ vertreten. Die Tab. 5 gibt einen Überblick über die identifizierten Publikationen, welche eine inhaltliche Analyse vornahmen.

**Table 5:** 

Art der Publikation		Anzahl der Publikationen	Prozent
Rekrutierung über Social Media		43	40,5
Inhaltsanalysen^a^ („Content analysis“)	Insgesamt	42	39,6
	Plattform	Youtube: 20/42	–
		Twitter: 11/42	
		Facebook: 5/42	
		Instagram: 2/42	
	Zielgruppe	Patient*innen: 16/42	–
		Medizinisches Fachpersonal: 7/42	
	Erkrankung	COVID-19: 7/42	–
		Rheumatoide Arthritis: 5/42	
		Spondyloarthritis: 3/42	
		Gicht: 3/42	
		Juvenile idiopathische Arthritis: 2/42	
		Systemischer Lupus erythematodes: 2/42	
		Psoriasisarthritis: 1/42	
		Vaskulitis: 1/42	
		Sjögren-Syndrom: 1/42	
		Fibromyalgie: 1/42	
Untersuchungen zum Einfluss von Social Media		9	8,4
Reviews		13	12,2
Gesamtzahl		106	100

^a^Mehrfache Nennungen möglich

## Chancen von Social Media

### Professionelle Netzwerke und Werbung

Soziale Netzwerke können im beruflichen Kontext insbesondere auch zur Vernetzung und zum Austausch mit Fachkolleg*innen genutzt werden. Für professionelles Netzwerken unter den Ärzt*innen wurden bisher meist soziale Netzwerke genutzt, die exklusiv erst nach Autorisierung zugänglich sind [12]. Diese Plattformen werden meist zur Diskussion verschiedener fachlicher Themen sowie für digitale Lehrformate verwendet. Als Beispiele solcher professioneller Netzwerke für Ärzte sind zu nennen: Sermo, Doximity, the Medical Directors Forum, Doctors Hangout, Doc2Doc oder Student Doctor Network [6].

Über diese rein berufsstandspezifischen Netzwerke hinaus ist in den letzten Jahren LinkedIn als professionelles Netzwerk und Jobplattform im beruflichen Kontext sehr populär geworden [6]. Verschiedene soziale Medien veröffentlichen ebenfalls Jobangebote und weisen auf Karriere- und Weiterbildungsmöglichkeiten hin. Je besser Nutzer*innen vernetzt sind, desto mehr entsprechende Angebote werden ihnen angezeigt [7]. Firmen und Gesundheitseinrichtungen nutzen diese Option, um immer passgenauer entsprechende Berufsgruppen mit ihren Werbe- oder Imagekampagnen anzusprechen.

### Education

Als Informations- und Wissensquelle hat das Internet rasant an Popularität gewonnen. Insbesondere durch die Pandemiesituation sind auch Online-Fortbildungsformate über Social Media deutlich in den Vordergrund gerückt.

Von Bedeutung sind insbesondere videobasierte Wissensformate. Hier kommt u. a. der Plattform YouTube eine besondere Rolle zu. Auch im Kontext von rheumatologischen Themen erhält die Plattform immer größeren Zulauf [8–10]. In mehreren aktuellen Studien wurde die Qualität von Informationen aus YouTube-Videos analysiert. Hierbei zeigte sich, dass die meisten Videos im Kontext von rheumatologischen Themengebieten einen hohen Informationsgehalt sowohl für medizinisches Fachpersonal als auch für Patient*innen vorweisen. Weiterhin existieren sowohl zu einzelnen rheumatologischen Erkrankungen als auch zu medikamentösen Therapieoptionen zahlreiche qualitativ hochwertige Videos auf der Plattform [8–10]. Ähnlich spielt auch Instagram eine immer größere Rolle in der Wissensvermittlung in der Rheumatologie. Hier zeigte eine jüngste Analyse rheumatologischer Inhalte der Plattform, dass Videoinhalte signifikant mit einer höheren Anzahl an Likes korrelierten [11].

Auch für Studierende gewinnen digitale Lehrangebote über Social Media immer mehr an Bedeutung. Während in der Pandemiesituation Präsenzveranstaltungen kaum noch möglich waren, profitiert auch die Rheumatologie von digitalen Lehrangeboten [12]. Die Attraktivität dieser Angebote besteht v. a. auch in ihrer Aktualität und Niederschwelligkeit: Informationen können deutlich schneller (und meist unkomplizierter) als in klassischen Lehrbüchern aktualisiert werden. Auch werden digitale Lehrinhalte immer leichter und häufig kostenfrei zugänglich, was die Wissensvermittlung unabhängig vom sozioökonomischen Hintergrund deutlich erleichtert [13]. Inzwischen entstehen auch eigene Plattformen für die medizinische Videofortbildung wie beispielsweise streamedup!. Bei diesem kommerziellen Anbieter erhalten Personen aus medizinischen Fachkreisen nach Registrierung Zugang zu teilweise kostenfreien, aber auch kostenpflichtigen Inhalten. Zu den Sponsoren des Unternehmens zählen mehrheitlich Unternehmen der pharmazeutischen Industrie, die auch die Möglichkeit haben, als solche gekennzeichnete Sponsoreninhalte über die Plattformen zu veröffentlichen.

Neben den klassischen Bild- und Tonangeboten in Form von Lehrvideos oder Podcasts erfreuen sich auch Infographiken und Bilderrätsel („image of the week“) z. B. auf Instagram oder sog. *Tweetorials* (kurze Lehrsequenzen auf Twitter) einer wachsenden Beliebtheit.

### Awarenesskampagnen

Die Verbreitung von Informationen über soziale Medien stellt auch eine Schlüsselmöglichkeit dar, um das Bewusstsein für bestimmte Erkrankungen und Situationen (z. B. COVID-19) zu erhöhen und die Aufmerksamkeit der Bevölkerung gezielt zu lenken [14]. Insbesondere in Pandemiezeiten können neben klassischen Medien wie Print und Funk die sozialen Medien genutzt werden, um eine breite Nutzer*innenschaft über aktuelle Zahlen, Entwicklungen und politische Entscheidungen zu informieren [5, 15]. Erfolgreiche Gesundheitskampagnen wurden bereits mithilfe von Social Media durchgeführt, um über Erkrankungen wie Depressionen [16], Krebserkrankungen [17] und die Prävention von Infektionen [18] aufzuklären bzw. zu sensibilisieren.

Über die verschiedenen Kanäle können einzelne Bevölkerungsgruppen durch auf sie zugeschnittene Inhalte und Formate gezielt angesprochen werden. Inzwischen haben auch staatliche Institutionen dies erkannt und verwenden vermehrt auch die Plattformen mit jüngerer Nutzer*innenschaft, wie z. B. Snapchat oder TikTok, um über Infektionsprävention und psychische Gesundheit aufzuklären [18].

Auch in sozialen Medien dienen qualitativ hochwertige Informationskampagnen als Gegengewicht, um Falschinformationen zu berichtigen bzw. Leser*innen gezielt auf valide Quellen aufmerksam zu machen [19].

### Crowdsourcing/Patientenbeteiligung/-interaktion

Als „digital crowdsourcing“ („crowd“: Gruppe, „to source“: beziehen) wird die internetbasierte, systematische Verteilung von Aufgaben auf eine Gruppe von digitalen Nutzer*innen bezeichnet [20]. Konzeptuell steht die Verlagerung einer Aufgabe weg von einem Individuum hin zu einer Aufteilung auf eine Gruppe im Vordergrund. Hierbei geht es um die kollektive Nutzung der Ressourcen und Erfahrungen einer beliebig großen Gruppe bei der Bewältigung von Aufgaben. Wie die systematische Analyse zeigt, ist die Rekrutierung von Patient*innen bzw. Ärzt*innen für wissenschaftliche Vorhaben inzwischen ein weit verbreitetes Phänomen (Tab. 5). Aktuelle Beispiele sind das *covid19-rheuma Register *sowie das *Covid-19 Impfregister *der Deutschen Gesellschaft für Rheumatologie (DGRh) und der Justus-Liebig-Universität Gießen [21]. Auf ersterer Plattform können Rheumatolog*innen Daten rheumatologischer Patient*innen mit COVID-19-Infektion erfassen, und COVID-19-positive Patient*innen können sich darüber hinaus aktiv via Hotline dort melden. Auf letzterer Plattform können u. a. Patient*innen mit rheumatologischen Erkrankungen auch selbst einen Fragebogen zur Impfung ausfüllen. Mittels dieser Crowdsourcing-Anwendung konnten in kurzer Zeit hohe Fallzahlen erreicht werden – mit Stand vom 12.01.2022 von beispielsweise 3635 Fällen im *covid19-rheuma Register* [22].

Eine ähnliche Initiative stellt das internationale Register der *COVID-19 Global Rheumatology Alliance *dar, in das auch die Daten des deutschen Registers einfließen [23]. Die genannten Plattformen zeigen exemplarisch, dass digitales Crowdsourcing eine rasche Datenerfassung mit Überwindung lokaler oder nationaler Grenzen ermöglicht.

Auch im Bereich von Social Media findet „digital crowdsourcing“ eine breite Anwendung. Beispielsweise werden Plattformen wie *Twitter* systematisch von *Medical Influencern* genutzt, um z. B. Pre-print-Publikationen zu diskutieren oder Fragebögen zu „patient reported outcomes“ (PRO) in andere Sprachen zu übersetzen. Zusätzlich können Patient*innen digital für die Teilnahme an Studien rekrutiert werden und ihre Daten „spenden“. Die zunehmende Präsenz von Patient*innen auf Social-Media-Plattformen ermöglicht eine einmalige Erreichbarkeit für Studienzwecke. Innerhalb weniger Stunden können so mehrere Hundert Patienten eingeladen und erfolgreich zur Teilnahme an Umfragen oder Registern motiviert werden [24]. Mittels Online-Umfragen können effizient neue Erkenntnisse und Stimmungsbilder gesammelt werden [25]. Insbesondere bei seltenen Erkrankungen können so über geografische Grenzen hinweg Befragungen durchgeführt werden [26]. Aber auch die breite Erreichbarkeit von Ärzt*innen in den Social Media birgt Potenzial. So können beispielsweise Interessent*innen für wissenschaftliche Kooperationen über Social Media gewonnen werden (vgl. hierzu z. B. [27, 28]).

Interessanterweise können Social-Media-Daten selbst als Quelle für wissenschaftliche Analysen zu bestimmten Fragestellungen im rheumatologischen Kontext verwendet werden. So spiegelt u. a. das Nutzer*innenverhalten gewisse Trends und Einstellungen der Patient*innen z. B. bezüglich der Einstellung zu Therapieoptionen [29] oder Ernährungsgewohnheiten wider [30]. Während durch die breite Zugänglichkeit zu Social Media ohne hohen ökonomischen Aufwand oder räumliche Beschränkung einerseits mehr Menschen erreicht werden können, besteht hier andererseits ein Selektionsbias für eine technologieaffine, oft eher jüngere Gruppe.

### Wissenschaftskommunikation an Laienpublikum und in Fachkreisen

Die Verbreitung von wissenschaftlichen Erkenntnissen ist essenziell für den Erfolg einer wissenschaftlichen Arbeit. Die Relevanz guter Wissenschaftskommunikation für ein breites Publikum belegt auch der „Communicator-Preis“, mit dem die Deutsche Forschungsgemeinschaft seit dem Jahr 2000 herausragende Leistungen auf diesem Gebiet honoriert [31]. Erneut wird hier der Kommunikation über Social-Media-Kanäle eine große Rolle zuteil. Ein prominentes Beispiel ist u. a. die Chemikerin und Wissenschaftsjournalistin Mai Thi Nguyen-Kim, die mit ihrem YouTube-Kanal *maiLab* einem Millionenpublikum wissenschaftliche Themen und Forschungsergebnisse näherbringt [32]. Hierfür wurde das Format u. a. mit dem Grimme Online Award in der Kategorie Wissen und Bildung ausgezeichnet.

Darüber hinaus gewinnt die Nutzung von Social Media auch innerhalb der wissenschaftlichen Community zur Diskussion von neuen Erkenntnissen oder zur Promotion eigener Forschungsergebnisse an Bedeutung [33, 34]. Fast ausnahmslos alle großen wissenschaftlichen Journale sind auf Social-Media-Plattformen präsent (vgl. Tab. 4). Um dieser Entwicklung Rechnung zu tragen, haben erste Journale (z. B*. Annals of Rheumatic Diseases/RMD Open)* eigene Social-Media-Editor*innen.

Weiterhin konnte bereits gezeigt werden, dass die Nutzung von Social-Media-Kanälen für die Verbreitung und Diskussion eigener Forschungsergebnisse die Zitationswahrscheinlichkeit von immunologischen und rheumatologischen Fachartikeln nachweislich erhöht [33, 34]. Des Weiteren werden in digitalen Journalclubs, wie z. B. dem EULAR-EMEUNET Journal Club auf Twitter, regelmäßig aktuelle Paper analysiert und mit den Autoren diskutiert.

Insbesondere im Rahmen von wissenschaftlichen Konferenzen und Meetings setzen viele Teilnehmer*innen auf die Verbreitung von Kongressinhalten über soziale Medien. Dies erhöht die Reichweite der entsprechenden Inhalte und ermöglicht eine ortsungebundene Diskussion über den Kongress hinaus [35]. So haben auch beim DGRh-Kongress 2021 Rheumatolog*innen v. a. auf Twitter unter dem *Hashtag #DGRh2021* live berichtet. Über den Account der AG Junge Rheumatologie wurden viele der kongressbezogenen Beiträge geteilt (s. auch Abb. 2). Gerade bei einer Vielzahl teils paralleler Kongressangebote können Follower*innen so einen Eindruck weiterer Inhalte und Diskurse gewinnen. Vor einem solchen Hintergrund hat auch das American College of Rheumatology beim ersten virtuellen ACR-Kongress im Jahr 2020 gezielt Medical Influencer als Twitter-Botschafter (ACR Twitter Ambassador) gewonnen, um die Bekanntheit der Konferenz zu erhöhen und die Konversationen darüber zu beleben.

## Risiken/Schwierigkeiten

### Verzerrung der Realität

Der niederschwellige Zugang zu sozialen Medien und die einfache Verfügbarkeit bringen jedoch auch Schwierigkeiten mit sich, da es hier meist keine übergeordneten Kontrollinstanzen gibt. Der Erfolg von Nachrichten oder Kampagnen wird über deren Verbreitungs- und Interaktionshäufigkeit (*Likes, Shares, Comments, Retweets etc*.) gemessen. Dies hat jedoch leider auch zur Folge, dass eine Vereinfachungstendenz, gezielte Emotionalisierung bis hin zur bewussten Skandalisierung von bestimmten Inhalten weiter zunimmt [36].

Insbesondere Kurznachrichtenformate, wie z. B. Twitter, erzwingen eine Verknappung von Nachrichteninhalten, was v. a. in der Kommunikation von Studienergebnissen potenzielle Vereinfachungen oder sogar Fehlinterpretationen begünstigen kann [37].

Der Begriff der „Fake News“ im Zusammenhang mit Social-Media-Nutzung hat sich mittlerweile im Sprachgebrauch fest etabliert. Insbesondere während der Corona-Pandemie wurde durch bewusste oder unbewusste Fehlinformation z. B. zu Medikamenten wie Hydroxychloroquin oder der Schutzimpfung gegen SARS-CoV‑2 vielerorts ungünstig Einfluss auf die Meinungsbildung vieler Menschen genommen. Die Anonymität des Einzelnen im Internet und die eingeschränkten rechtlichen Möglichkeiten, gegen Fehlinformationen in Social Media vorzugehen, begünstigen diese Effekte [37].

In diesem Zusammenhang hat sich auch die Methode des *Clickbaitings *etabliert. Hier wird mittels einer reißerischen Überschrift der*die Nutzer*in dazu animiert, Angebote oder Webseiten zu öffnen. Dem*der Leser*in wird gerade ausreichend Information geboten, um seine*ihre Neugier zu wecken, aber nicht genügend, um diese auch zu befriedigen.

Eine weitere künstliche Verzerrung der Inhaltsreichweite von Social Media besteht durch die Nutzung von sog. *Social Media Bots*. Dabei handelt es sich um Computeralgorithmen, die in den sozialen Netzwerken menschliche Verhaltensmuster und menschliche Präsenz simulieren. Hierdurch wird künstlich Interesse suggeriert bzw. eine künstliche Meinungsmehrheit hergestellt. Dieser Effekt kann von Unternehmen oder Interessengemeinschaften genutzt werden, indem diese für ein gewünschtes Verhalten solcher Bots bezahlen.

### Distanzverlust

Mit der Verwendung sozialer Medien wird die klassische Beziehung zwischen Ärzt*innen und Patient*innen auf die Probe gestellt. Online-Plattformen vereinfachen den Zugang zum*zur behandelnden Ärzt*in und erleichtern die Kommunikation. Es sollte jedoch streng darauf geachtet werden, dass hierbei Professionalität und Abstand gewahrt werden und die Kommunikation weiterhin einen formalen Charakter behält [38]. Vorab sollte klargestellt werden, in welchen Situationen eine Kommunikation über soziale Medien möglich ist, und auch die Grenzen (keine Verfügbarkeit rund um die Uhr, kein Ersatz der Sprechstunde) sollten vorab festgelegt werden. Ein Statement des American College of Physicians und der Federation of State Medical Boards warnt Ärzt*innen zudem davor, „Freundschaftsanfragen“ von Patient*innen auf sozialen Plattformen zu akzeptieren [38]. Grundsätzlich gilt es, das Vertrauensverhältnis zwischen Ärzt*innen und Patient*innen zu wahren. Dies kann besonders strapaziert werden, wenn online Informationen über den jeweils anderen gesucht oder bei erfolgter Vernetzung vermehrt angezeigt werden [39]. Dies sollte sich auch jede*r Ärzt*in verdeutlichen, bevor Meinungen und Ansichten in den sozialen Medien verbreitet werden.

Trotz Online-Angeboten zur Patient*innenschulung und der Möglichkeit, persönliche, individuelle Fragen zu klären, ersetzen die sozialen Medien nicht den beziehungs- und vertrauensstiftenden Wert eines persönlichen Kontaktes weder unter Patient*innen oder Ärzt*innen noch zwischen Ärzt*in und Patient*in.

### Datenschutz/Privatsphäre/Copyright

Bei fehlerhafter Nutzung von Social Media können sich auch datenschutzrechtliche Verletzungen der Privatsphäre oder Verstöße gegen Copyright ergeben. So sollte man über Social Media keinesfalls patientensensitive Daten teilen. Fallberichte aus dem klinischen Alltag oder Patient*innenfotos sollten auch nicht ohne ausdrückliche schriftliche Zusicherung der Patient*innen über Social Media veröffentlicht werden. Weiterhin ist beim Teilen von Kongressbeiträgen oder von Publikationen darauf zu achten, dass die jeweiligen Autor*innen oder Verlage mit der Veröffentlichung der Daten einverstanden sind.

Weiterhin gilt es, bei der Einstellung seiner jeweiligen Social Media Accounts darauf zu achten, welche Inhalte für wen sichtbar sind, und nach Möglichkeit private Meinung von beruflichen Aufgaben und Ämtern zu trennen bzw. seine Aussagen entsprechend zu kennzeichnen. Anhand des Interaktionsprofils lassen sich viele persönliche Neigungen und Interessen (z. B. politische Orientierung) ableiten, die u. U. im Konflikt mit beruflichen Aufgaben und Funktionen stehen können. Die Bundesärztekammer hat 2014 in einer Handreichung mit dem Titel „Ärzte in Sozialen Medien“ diese und weitere Punkte mit Fallbeispielen und konkreten Empfehlungen für eine Verwendung im Einklang mit dem Berufsrecht adressiert [40]. Ein Exzerpt in Form allgemeiner Regeln für Ärzt*innen im Umgang mit Social Media gibt Infobox 1 wieder.

### Zeitaufwand

Die sozialen Medien unterliegen einer extremen Dynamik. Bilder und Nachrichten werden teilweise innerhalb von Minuten kommentiert, geteilt und teilweise auch verfremdet. Je nach Kontext und Inhalt kann ein Millionenpublikum in Echtzeit an einem digitalen Diskurs teilnehmen. Um hier am Puls der Zeit zu bleiben, ist jedoch ein vermehrter Zeitaufwand notwendig. Eine Studie von Twenge et al. zeigte, dass Schüler*innen der 12. Klasse bis zu 6 h am Tag im Jahr 2016 in den sozialen Medien verbrachten, wobei diese Zeitspanne tendenziell zunahm; 82 % nutzten täglich soziale Medien [41].

Durchschnittlich verbrachten Erwachsene weltweit zwischen 2012 und 2020 mehr als 2 h täglich in sozialen Medien [42]. Hier ist eine individuelle Kosten-Nutzen-Abwägung bezüglich des zeitlichen Aufwands der Social-Media-Nutzung sicherlich wichtig für den Einzelnen [43]. Diesbezüglich kann es hilfreich sein, private und berufliche Accounts zu trennen, um möglichst effizient zu sein [44].

## Zusammenfassung/Ausblick

Social-Media-Plattformen werden außerhalb des privaten zunehmend auch im beruflichen Kontext relevant. Diese Entwicklung wird getragen durch eine nachwachsende Generation von *Digital Natives*, die die verschiedenen Plattformen alltäglich verwenden und auch immer stärker als primäre Informationsquelle nutzen. In diesem Zusammenhang ist auch eine teilweise Verlagerung des akademischen und gesellschaftlichen Diskurses zu Gesundheitsthemen auf digitale Plattformen zu beobachten.

Vor allem Twitter kommt dabei aktuell für den wissenschaftlichen Diskurs eine führende Rolle zu, aber auch andere Netzwerke werden vermehrt je nach Kontext und Zielgruppe für rheumatologische Themen verwendet. Über digitale Informations- und Crowdsourcing-Kampagnen können Lehr- und Aufklärungsprojekte sowie Forschungskampagnen zielgruppenspezifisch (auch über Ländergrenzen hinweg) direkt an ein breites Publikum herangetragen werden. Durch die digitale Vernetzung kann der ortsungebundene fachliche Austausch erleichtert werden.

Trotz vieler positiver Aspekte ist jedoch auch ein kritischer Umgang mit sozialen Medien und der Interaktion in den Netzwerken zu wahren. So sind insbesondere Fehlinformationen und eine Informationsverknappungs- und Skandalisierungstendenz kritisch zu betrachten. Darüber hinaus sind datenschutzrechtliche Regularien dringend zu berücksichtigen, sowie eine eindeutige Trennung von privatem und beruflichem Auftreten ist zu empfehlen. Diese Punkte sollten allerdings nicht dazu führen, dass Rheumatolog*innen sich der Nutzung sozialer Medien grundsätzlich verwehren. Vielmehr muss daraus eine differenzierte Auseinandersetzung mit dem Thema auch in Lehre, Weiterbildung und wissenschaftlichem Diskurs erwachsen, um den stetigen Einzug der sozialen Medien in unseren Berufsalltag mitzugestalten.

Speziell für die Rheumatologie bietet sich durch Präsenz in den sozialen Medien die Chance, die breite Öffentlichkeit einerseits und Nachwuchsmediziner*innen andererseits auf sich aufmerksam zu machen und das Bild des Faches zu prägen. Bei dieser Aufgabe können wir uns auf 2 wesentliche Attribute der modernen Rheumatologie besinnen: Innovationsgeist und die Wertschätzung guter Kommunikation.

### Infobox 1 10 Regeln für Ärzt*innen im Umgang mit sozialen Medien

- Ärztliche Schweigepflicht beachten
- Keine Kolleg*innen diffamieren – Netiquette beachten
- Berufliches und privates Profil voneinander trennen
- Grenzen des Arzt*innen-Patient*innen-Verhältnisses nicht überschreiten
- Fernbehandlungsverbot beachten
- Keine berufswidrige Werbung über soziale Medien
- Datenschutz und Datensicherheit beachten
- Selbstoffenbarung von Patient*innen verhindern
- Zurückhaltung bei produktbezogenen Aussagen
- Haftpflichtversicherung checken

## Supplementary Information




