# Supplementary material for: Social media—Chances and risks for rheumatology
Source: Z Rheumatol. 2022 Apr 8;81(5):413–22. [Article in German] doi: 10.1007/s00393-022-01201-9 (PMC8990654; doi:10.1007/s00393-022-01201-9)
Supplement: Supplementary file 1 [file 393_2022_1201_MOESM1_ESM.pdf]

Zusatzmaterial

Die Grundlage der vorliegenden Publikation bildet eine systematische Literaturrecherche zur Identifikation rheumatologischer Themen im Zusammenhang mit der Nutzung von Social Media.

Suchstrategie:

Zur Identifikation von Publikationen, welche die Anwendung und Nutzung von Social Media in der Rheumatologie untersuchten, wurde die medizinische Literaturdatenbank MEDLINE (via PubMed) durchsucht. Suchbegriffe zur Identifikation von Social Media Inhalten waren: „social media“, „facebook“, „instagram“, „twitter“, „youtube“, „linkedin“ und „researchgate“. Suchbegriffe zur Identifikation von rheumatologischen Themen bzw. Erkrankungen waren: „rheumatology“, „arthritis“, „vasculitis“, „connective tissue disease“, „CTD“, „lupus“, „systemic sclerosis“, „scleroderma“, „myositis“ „sjogren“, „psoriasis“, „spondyloarthritis“, „gout“ und „rheumatic“. Die Suchbegriffe wurden für Titel und Abstract verwendet.

("rheumatology"[Title/Abstract] OR "arthritis"[Title/Abstract] OR "vasculitis"[Title/Abstract] OR "connective tissue disease"[Title/Abstract] OR "CTD"[Title/Abstract] OR "lupus"[Title/Abstract] OR "systemic sclerosis"[Title/Abstract] OR "scleroderma"[Title/Abstract] OR "myositis"[Title/Abstract] OR "sjogren"[Title/Abstract] OR "psoriasis"[Title/Abstract] OR "spondyloarthritis"[Title/Abstract] OR "gout"[Title/Abstract] OR "rheumatic"[Title/Abstract]) AND ("social media"[Title/Abstract] OR "facebook"[Title/Abstract] OR "twitter"[Title/Abstract] OR "instagram"[Title/Abstract] OR "youtube"[Title/Abstract] OR "linkedin"[Title/Abstract] OR "researchgate"[Title/Abstract])

Einschlusskriterien

- Publikation adressiert rheumatologische Erkrankungen/Themen UND
    - Publikation untersucht Inhalte von Social Media (z.B. content analysis)
    - Publikation ist ein Übersichtsartikel zu Social Media
    - Aufruf zur Studienteilnahme über Social Media Studien

Ausschlusskriterien

- Abstract nicht verfügbar
- Studienprotokolle
- Erkrankung: nicht-rheumatologische Erkrankung/Themen
- Literatursuche über ResearchGate
- Publikationen mit ausschließlich Psoriasis
